# “Mischievous Uncles” as Rule Breakers: Intersectional Stereotypes and Risk Perceptions During the Coronavirus Pandemic in Turkey

**DOI:** 10.1177/2056305120949268

**Published:** 2020-08-11

**Authors:** Didem Türkoğlu, Meltem Odabaş

**Affiliations:** 1 New York University Abu Dhabi, United Arab Emirates; 2 Indiana University Bloomington, USA

**Keywords:** COVID-19, intersectionality, age, class, and gender, Turkey, Twitter and news media

## Abstract

Responses to crises can highlight and exacerbate class inequalities. Seemingly neutral policy measures taken during the COVID-19 pandemic that aim to protect high-risk groups can lead to a shift in the public discourse that deprives citizens of their agency based not only on their age but also their class. In this article, we focus on the case of Turkey, one of the countries with the fastest growth of novel coronavirus cases in late March 2020, where the government introduced a curfew for people over the age of 65, while actively encouraging the rest of the working-age population to stay at home. An intersectional analysis of the Twitter campaign #StayatHome (#*EvdeKal*) and the media outlets’ news coverage of the policy implementation show that both platforms circulated human-interest stories of working-class men who defy the curfew predominantly. Both the stories and Twitter user comments often defined the subjects of those stories as rule-breakers and, therefore, as “mischievous uncles.” They became the scapegoats, while upper and middle classes avoided the label. These findings have implications for the framing of policy outcomes and welfare provisions as well as oppositional politics that push for the expansion of labor protections during the pandemic.

## Introduction

While many studies highlight the effect of power structures that lie at the intersection of gender and the construction of the public sphere on social media (Lutzky & Lawson, 2019), the intersection of ageism and its class dimension is often overlooked. And yet, the discourse that deprives the elderly of their agency has severe consequences for policy outcomes (Calasanti, 2020). Moreover, extant research shows that responses to crises can highlight and exacerbate class inequalities (Hartman et al., 2006). Furthermore, social media such as Twitter take an increasingly more prominent role in crisis communication (Spence et al., 2015) in interaction with traditional media outlets. However, working-class organizations are represented significantly less on Twitter (Schradie, 2019). Given these differences in power relations, as policies and media narratives focused substantially on the elderly during the COVID-19 pandemic, how do age, gender, and class intersect?

Our analysis focuses on the case of Turkey, one of the countries with the fastest growth of novel coronavirus cases in late March 2020. The government initially introduced a curfew for people over the age of 65, while actively encouraging the working-age population to stay at home without a nation-wide quarantine. An intersectional analysis of the Twitter campaign #StayatHome (#*EvdeKal*) and media outlets’ news coverage about the policy implementation show that media outlets’ human-interest stories often depicted the working-class men who defy the curfew as “irresponsible citizens.” At the same time, middle and upper classes were able to avoid the labeling, and women strangely disappeared in the background. As a result, at the intersection of all these differences lie the “mischievous uncles.” This was a trope used in one of the most retweeted comments in our dataset, it captures news stories and Twitter users’ denial of agency agency to a segment of the population in their depiction of the social problems that arise with COVID-19.

## Curfew for the Elderly

When the first COVID-19 case was announced on 10 March 2020 in Turkey, media campaigns emerged to encourage people to stay at home and avoid public gatherings and places as much as possible, similar to many countries. TV stations, newspapers, and social media covered calls to remain at home widely. Users on Twitter chose to #StayAtHome to participate in the campaign to “flatten the curve.” As the number of COVID cases in Turkey quickly rose (confirmed cases doubled every day during the first 10 days since the first case was announced; see Ahn, 2020 for details), public demand for a curfew also increased.

The president announced the strict lockdown for the citizens over the age of 65 on 21 March. Elderly citizens were not allowed to leave home under any circumstances except for hospital visits. Not surprisingly, responses to the pandemic measures reflected the political divide in Turkey. Pro-government media outlets gave their full support to the government, which opposed a blanket curfew even in the cities that were hit the hardest. Left-wing/oppositional media in tandem with opposition parties demanded a nation-wide lockdown. While both sides of the political spectrum endorsed the curfew for 65+, they remained polarized regarding the expansion and implementation of such measures. Nevertheless, these discussions reflected the power relations at the intersection of class and gender.

“Mischievous uncles” soon became a trope for the violators of the lockdown. Although Turkish has no gender differentiation in the third-person pronoun, and the language of the policy was also gender-neutral, the tweets often used gendered nouns to refer to the elderly. In the Turkish language, informal qualifiers such as uncle or auntie are often used to refer to the elderly men and women, regardless of kinship ties, which reflect the level of affection. Yet, there is also a status distinction in these affectionate qualifiers. In the absence of familial or in-person connections, these words commonly refer to older adults of lower social status. For those who enjoy higher status, as perceived by the speaker, the appropriate qualifier would be sir or lady. Combined with the material conditions of the news stories (e.g., using public transport or financial need to work), such linguistic differentiation highlights the discursive and material inequalities.

## News Stories: Working-Class and the Poor as Rule-Breakers

**Figure 1.**
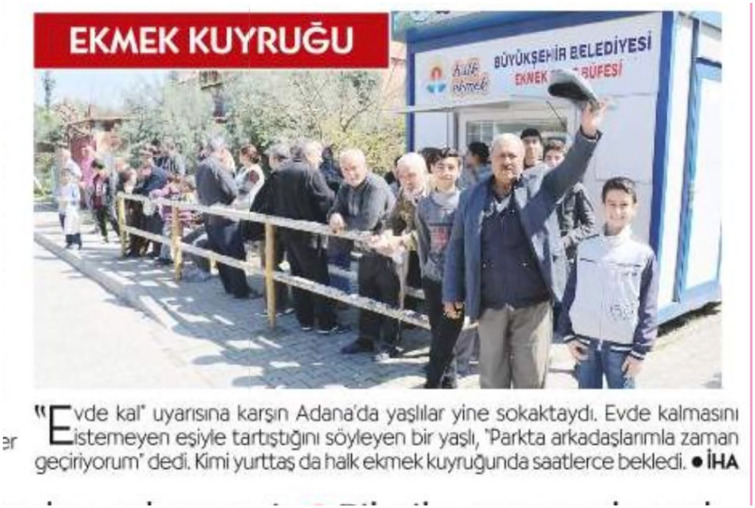
The news piece reads, “The elderly were again on the street in Adana despite the warnings to stay at home. An elderly who told [the reporter] that they argued with their partner about not wanting them to sit at home said the following, ‘I am passing the time at the park with my friends.’ Some citizens waited for hours to get subsidized bread” (Cumhuriyet, 22nd March 2020).

News about the curfew included several human-interest stories, both in the mainstream news media and the alternative/oppositional media outlets. A keyword search conducted in news archives show two predominant tropes. News stories included cases where citizens warned those who violated the curfew or reporters interviewing elderly citizens who defended themselves for not abiding by the restrictions.^1^ For example, *Hürriyet*, a pro-government newspaper with one of the highest circulation numbers, ran a story of a local reporter in Amasya, an agricultural city. The reporter, holding a microphone from her balcony warns an elderly man on the street: “Uncle Mehmet, aren’t you 65 years old? Why are you on the street uncle? Let’s abide by the rules!”^2^ Similarly, *Hürriyet* ran stories about violations in Kasımpaşa, one of the more impoverished neighborhoods in İstanbul.^3^ *Sabah*, another pro-government outlet ran similar stories. One included an old woman that could not get on the bus in Zonguldak, a mining city in northern Turkey, due to the curfew. Similar to the above cases, the reporter asks, “Auntie, how old are you” as she tries to get on the bus.^4^ Even though the news stories refer to the elderly in a neutral language, a photo could tell the story of working-class/poor people, often men. Figure 1 shows the people waiting on the line to get subsidized bread, reported by the left-wing *Cumhuriyet*.

Following this narrative of rule-breakers, soon videos of harassment emerged on social media until a backlash stoped them. In a video from the capital city, Ankara, a young man pretended to be a policeman, while an older man tried to explain that he went to the hospital and was on his way home.^5^ This video led to public outrage and started a backlash against labeling the elderly. As a response, the ministers of health, interior, and justice all tweeted condemning the man who took the video,^6^ which brought fame to “Uncle İhsan.” His story as a working-class man who weaved carpets for over 30 years in a 10-square-meter workshop was covered widely by left-wing outlets^7^ and both the right-wing and the left-wing media outlets covered his message to stay at home.^8^ While the news media’s coverage underlined the class dimension with a tendency to cover the stories of older men, this gendered pattern has become much more visible on social media.

## Gendered Patterns on Twitter

Twitter is a popular platform in Turkey: In March 2020, Twitter accounted for about 20% of the web activity in Turkey, putting it on par with Instagram.^9^ We used Twitter’s standard search and the *rtweet* package in R to stream tweets that include a list of coronavirus related hashtags^10^ in real-time, during 16 March–21 April 2020. In this period, the discussions around how the elderly were affected by the pandemic increased. This increase was triggered by the news items that indicate that people who are above the age of 65 are the most vulnerable. However, the discussion in the social media took a decisively gendered pattern.

**Figure 2.**
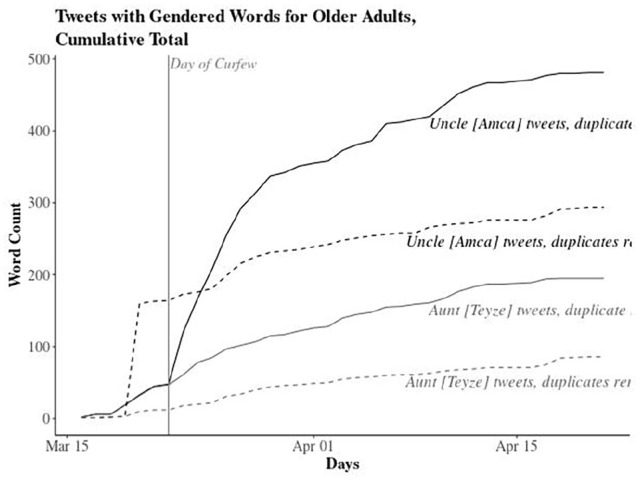
Cumulative count of #StayAtHome tweets that include gendered qualifiers for the elderly.

To highlight this gendered pattern, we further analyzed the tweets that specifically focused on the elderly. We filtered tweets that include the Turkish-language hashtag #stayathome (*#evdekal*) (136,632 tweets) to analyze the development of public opinion about the curfew on Twitter. Figure 2 shows that the discussion had a gendered pattern since its beginning but it became much more pronounced after the introduction of the curfew.

In the first phase of the discussion, before the curfew was introduced, some users demanded a curfew for the elderly. Figure 2 demonstrates the total number of tweets (in bold) as well as the number of unique tweets once the duplicates (like retweets) are removed. The day of curfew indicates the day of the announcement of the curfew. In our sample, before the announcement of the curfew in the evening of 21 March, a majority of tweets that mentioned uncles or aunts complained about their unruly behavior. We then qualitatively analyzed the unique tweets.

One of the first tweets that referred to the uncles and aunts, asked the elderly that have just returned from the pilgrimage to Mecca to stay at home and follow self-isolation rules for those who recently returned from abroad:

. . . three full loads of buses, there are still people who don’t take this seriously. Especially pilgrim aunties, uncles . . . even if you don’t care what happens to you, don’t commit any sins by infecting others . . . #StayAtHomeTurkey.

**Figure 3.**
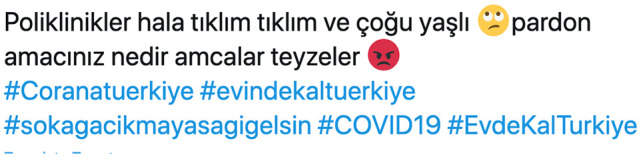
“Clinics are still overcrowded and most [of the people there] are elderly. Excuse me but what is your purpose, uncles and aunties?”

Similarly, some users argued that the unruly behavior of the elderly paved the way for the introduction of a nation-wide curfew. Tweets dating 17 March (4 days before the curfew) reflect this trend. While one complains about the unnecessary visits to clinics (see Figure 3), another points out that “the uncles and aunties queue in front of the banks to get their retirement checks despite the warnings to stay at home.”

**Figure 4.**
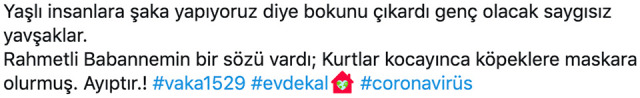
These disrespectful young slimes went too far with these pranks on old people. My late grandmother used to say; when the wolves get old the dogs make fun of them. What a shame!

With the introduction of the curfew and the human-interest stories on the news, the class dimension became much more visible. The working class/poor became the scapegoats, while upper and middle classes avoided the label as the news disproportionately narrated a class story. This class story became heavily gendered on Twitter as the stories of curfew violators trigger more discussion as depicted in Figure 2. For example, “Uncles are like mischievous kids, they don’t listen [to reason]” was retweeted frequently (36 times in our dataset) during 23–24 March 2020. Such tweets, as well as the publicized story of “Uncle İhsan,” triggered a backlash on Twitter as a response to those who target and condemn the elderly (see Figure 4).

As the trope backfired with the case of uncle İhsan and curfews were also introduced for people below the age of 20, references to “uncles” also stopped (see Figure 2).

## Conclusion

Both the news stories and Twitter user comments often define the subjects of curfew-violator stories as rule-breakers, as “mischievous uncles.” As the word implies a lower socio-economic status in the absence of personal connections, upper and middle classes avoid the label. In the early days of the curfew, the predominant narrative both in the mainstream media and on Twitter was to treat the elderly as “mischievous” children who do not know better and therefore need to be disciplined for their own good. The underlying assumption in this narrative was that if people were above 65 years old, they would not need to work anyways. The working class bears the most substantial burden as they were the ones who crammed in less than ideal housing situations or had to work. This situation has been a concern for the left-wing unions and needs to be recognized as the pandemic measures shape labor policies.

Discursively, the media coverage of the curfew exacerbated the inequalities at the intersection of class and age with its emphasis on the mischievous uncles. In online discussions, Twitter users depicted the working-class men as the rule-breakers. The silence around the women in the same platform was also noteworthy as it highlights the understanding of public spaces as predominantly the domain of the men. Shaming and excluding subsections of the population in this manner could potentially translate into the acceptance of more discriminatory policies. Our analysis focuses on the intersection of age, gender, and class, but there are, of course, more dimensions such as ethnicity and religion. In the light of our findings, we hope that future research focuses more on the different dimensions of intersectionality.

## Notes

1. https://www.hurriyet.com.tr/gundem/istanbulda-65-yas-ustu-sokaga-cikma-yasagina-uymadi-disariya-cikmayin-dediler-ama-41474824

2. https://www.hurriyet.com.tr/gundem/balkonundan-mikrofonla-sokaktakilere-65-yas-uyarisi-yapti-niye-sokaga-ciktin-amca-ya-41475504

3. https://www.hurriyet.com.tr/gundem/istanbulda-65-yas-ustu-sokaga-cikma-yasagina-uymadi-disariya-cikmayin-dediler-ama-41474824

4. https://www.sabah.com.tr/video/haber/son-dakika-turkiye-corona-virusu-yasagina-uymayan-yasli-teyzenin-bu-goruntulerini-konusuyor-video

5. https://www.sabah.com.tr/yasam/2020/03/24/son-dakika-corona-virusu-onlemleri-surerken-yasli-adami-videoya-aldi-gercek-ortaya-cikti-tepki-yagiyor

6. http://www.diken.com.tr/sokaga-cikan-80-yasindaki-vatandasla-dalga-gecip-videosunu-ceken-genc-gozaltinda/

7. https://www.birgun.net/haber/dalga-gecilen-ali-ihsan-yavasca-nin-hikayesi-30-yil-kilim-dokuyarak-6-cocuk-buyuttu-293183

8. https://www.milliyet.com.tr/gundem/ihsan-amca-ilk-kez-konustu-sikayetci-degilim-6173641 https://www.hurriyet.com.tr/gundem/ozur-dileriz-ihsan-amca-41477075 https://www.yenisafak.com/koronavirus/ozur-dileriz-ihsan-amca-suleyman-soylu-ile-telefonda-gorustu-3531206 https://www.trthaber.com/haber/gundem/ihsan-amcaya-o-saygisizligi-yapan-yakalandi-469733.html https://t24.com.tr/haber/tepki-ceken-videodaki-ihsan-amca-konustu-ceza-vermisler-ama-sikayetci-degilim,868651 https://halktv.com.tr/yasam/tepki-ceken-videodaki-ihsan-amca-ilk-kez-konustu-421860h

9. https://gs.statcounter.com/social-media-stats/all/turkey

10. The list of hashtags include the following: #coronavirus, #corona, #viruscorona, #koronavirus, #korona, #viruskorona, #covid19, #covid-19, #covid_19, #covid2019, #sarscov2, #sars-cov-2, and #sars_cov_2.
